# DEVELOPMENT AND VALIDATION OF A PREDICTION MODEL FOR SIX-MONTH SURVIVAL OF MEDICARE BENEFICIARIES IN ASSISTED LIVING

**DOI:** 10.1093/geroni/igae098.0515

**Published:** 2024-12-31

**Authors:** Andrew Huang, Laura Dionne, Ann Reddy, Peter Serina, Ellen McCreedy

**Affiliations:** Brown University, Providence, Rhode Island, United States; Brown University, Providence, Rhode Island, United States; Brown University School of Public Health, Providence, Rhode Island, United States; Brown University School of Public Health, Providence, Rhode Island, United States; Veterans’ Administration, Providence, Rhode Island, United States

## Abstract

Prediction of mortality risk is a prognostic tool for identifying who may benefit the most from timely and appropriate end-of-life care. However, few mortality risk prediction models have been developed for community-dwelling older adults in assisted living centers (ALCs). To estimate individual risk of all-cause mortality for Medicare beneficiaries in assisted living, we used data extracted from electronic health records (EHR) and linked with Medicare claims from 2015-2022 to develop and validate a multivariable logistic regression on the outcome of all-cause mortality occurring within six months. Eligible residents had at least one year of Medicare enrollment, six months of continuous Medicare fee-for-service and Part D drug coverage, and no history of hospice at the time of prediction. Candidate predictors included individual-level demographics, healthcare costs and utilization, and available clinical features, including diagnoses, medications, and assessments of physical and cognitive function. Final predictors were identified with stepwise selection procedures and expert opinion. A k-fold cross-validation scheme with stratified sampling was used and model performance was assessed on discrimination and calibration statistics. We identified 31,274 residents from 1,228 ALCs eligible for mortality risk prediction. At baseline, the mean (sd) age was 85.3 (7.7) years, 69.0% were female, and 87.2% identified as non-Hispanic White. Dementia was prevalent among 11.5% of residents and 6.3% of residents died within six months. The final model returned an AUROC of 0.74 and calibration plots showed consistency between predicted and observed mortality outcomes. This EHR and claims-based model may help residents in ALCs receive better targeted end-of-life care.

